# Effects of the COVID-19 Pandemic on the Decision and Doubts About Vaccination in Catalonia: Online Cross-sectional Questionnaire

**DOI:** 10.2196/41799

**Published:** 2023-03-06

**Authors:** Agnes Huguet-Feixa, Eva Artigues-Barberà, Joaquim Sol, Pere Godoy, Marta Ortega Bravo, MC-MUVA

**Affiliations:** 1 Institut Catatà de la Salut Primary Care Center Rambla Ferran Lleida Spain; 2 Research Support Unit Lleida Fundació Institut Universitari per a la recerca a l'Atenció Primària de Salut Jordi Gol i Gurina Lleida Spain; 3 Institut Català de la Salut Primary Care Center Balàfia-Pardinyes-Secà de Sant Pere Lleida Spain; 4 Faculty of Nursery and Physiotherapy University of Lleida Lleida Spain; 5 Institut de Recerca Biomèdica Department of Experimental Medicine University of Lleida Lleida Spain; 6 Institut Catatà de la Salut Primary Care Lleida Spain; 7 Hospital Universitario Santa Maria. Gestió de Serveis Sanitaris. Departament de Salut Lleida Spain; 8 Centro de Investigación Biomédica en Red de Epidemiologia y Salud Publica Instituto de Salud Carlos III Madrid Spain; 9 Institut de Recerca Biomèdica Applied Epidemiology Research Group University of Lleida Lleida Spain; 10 Faculty of Medicine University of Lleida Lleida Spain; 11 See Acknowledgments

**Keywords:** COVID-19, vaccines, vaccine hesitancy, anti-vaccine, decision-making, disease, questionnaire, electronic, pandemic, vaccination

## Abstract

**Background:**

Hesitancy to get vaccinated during the COVID-19 pandemic may decrease vaccination coverage and facilitate the occurrence of local or global outbreaks.

**Objective:**

The objective of this study was to analyze the impact of the COVID-19 pandemic in Catalonia on 3 aspects: the decision to get vaccinated against COVID-19, changes in opinion about vaccination in general, and the decision to get vaccinated against other diseases.

**Methods:**

We performed an observational study with the population of Catalonia aged 18 years or over, obtaining information through a self-completed questionnaire in electronic format. Differences between groups were determined using the chi-square test, Mann-Whitney *U* test, or the Student *t* test.

**Results:**

We analyzed the answers from 1188 respondents, of which 870 were women, 47.0% (558/1187) had sons or daughters under the age of 14 years, and 71.7% (852/1188) had studied at university. Regarding vaccination, 16.3% (193/1187) stated that they had refused a vaccine on some occasion, 76.3% (907/1188) totally agreed with vaccines, 1.9% (23/1188) were indifferent, and 3.5% (41/1188) and 1.2% (14/1188) slightly or totally disagreed with vaccination, respectively. As a result of the pandemic, 90.8% (1069/1177) stated that they would get vaccinated against COVID-19 when they are asked, while 9.2% (108/1177) stated the opposite. A greater intention to get vaccinated was observed among women; people older than 50 years; people without children under 15 years of age; people with beliefs, culture, or family in favor of vaccination; respondents who had not previously rejected other vaccines, were totally in favor of vaccines, or had not increased their doubts about vaccination; and respondents who had not changed their decision about vaccines as a result of the pandemic. Finally, 30.3% (359/1183) reported an increase in their doubts regarding vaccination, and 13.0% (154/1182) stated that they had changed their decision about routinely recommended vaccines as a result of the pandemic.

**Conclusions:**

The population studied was predominantly in favor of vaccination; however, the percentage of people specifically rejecting vaccination against COVID-19 was high. As a result of the pandemic, we detected an increase in doubts about vaccines. Although the final decision about vaccination did not primarily change, some of the respondents did change their opinion about routine vaccinations. This seed of doubt about vaccines may be worrisome as we aim to maintain high vaccination coverage.

## Introduction

Vaccination is the most effective way to prevent diseases. Currently, immunization prevents 2 million to 3 million deaths per year, and, if vaccination coverage improves, another 1.5 million deaths could be avoided [1]. A decrease in vaccination represents a threat to the collective immunity acquired in recent years thanks to the efforts of health professionals [2].

The COVID-19 pandemic has drastically affected people and health systems worldwide. It has become a catalyst for many scientific advances, including the conception of a vaccine against COVID-19 [3]. This vaccine is considered one of the most important instruments to limit the spread or eliminate the pandemic, but its success is related to its acceptance worldwide [4-6].

According to the definition proposed by MacDonald and the SAGE Working Group on Vaccine Hesitancy [7], vaccine hesitancy is situated on a continuum from acceptance of all vaccines to total rejection. People who are hesitant about vaccination represent a heterogeneous group between these 2 extremes.

In 2019, the World Health Organization (WHO) rated vaccination hesitancy caused by antivaccine movements as one of the top 10 threats to global health [8] and warned of the dramatic growth of antivaccine theses and “fake news” in western Europe [9,10]. Vaccine hesitancy can be fueled by health information obtained from a variety of sources, including new media like the internet and social media platforms [2,4,11-13]. In particular, social networks have become a new paradigm for medical care, where power has passed from health professionals to patients and the legitimacy of science is questioned [2,14].

The antivaccine infodemic (misinformation and unfounded rumors about infection and vaccination) increased during the COVID-19 pandemic. It appeared on social networks long before the arrival of an effective vaccine, eroding confidence in vaccination among the community and making the job of health professionals difficult [4,10,11,15-17].

Globally, 63.1% of the adult population has received one dose of the COVID-19 vaccine, and 55.7% has received the complete regimen [18]. At the beginning of 2022, the Spanish Ministry of Health published that, in Spain, 80.5% of the total population and 90.4% of the population older than 12 years had been fully vaccinated against COVID-19 [19]. On the same dates, the Statistical Institute of Catalonia published that, in this region, 79.5% of the total population and 85.4% of the population over 12 years of age had been fully vaccinated against COVID-19 [20,21].

In this context, our study sought to assess the impact of the pandemic on the decision to get vaccinated against COVID-19, on the possible increase in doubts about vaccines in general, and on the decision to get vaccinated against other diseases. We also wanted to describe the potential influence exerted by gender and sociocultural characteristics.

## Methods

### Study Design

The main objectives of the study were to assess the impact of the pandemic on public opinion about 3 aspects: the decision to get vaccinated against COVID-19, doubts about vaccinations in general, and the decision to get vaccinated against other diseases.

We performed an observational study in the population in Catalonia aged 18 years or older who had access to the online survey in 2021 and who had the faculty to decide on their or others’ vaccination.

We obtained the information through an electronic, self-administered questionnaire designed by the project research team. A pilot test was performed before the definitive questionnaire was obtained. Both were registered on a Research Electronic Data Capture (REDCap) web platform on a centralized server where the data remain in the custody of the Institut Català de la Salut. Through the REDCap web platform, we also built a database of the participants. Anonymous information was exported to the statistical packages used for subsequent analysis. The REDCap platform generated a link [22] for participation in the survey that was disseminated through scientific societies, social networks, research institutes, pediatricians, and nurses in primary care.

### Ethical Considerations

This study was approved by the ethics and clinical Research Committee of the Institut Universitari per a la recerca a l'Atenció Primària de Salut (IDIAP) Jordi Gol i Gurina, with code 20/221-P. The study was conducted in accordance with the principles of the Declaration of Helsinki. The variables collected were treated anonymously and to guarantee the confidentiality of the data, as established in Regulation (EU) 2016/679 of the European Parliament and the Council of April 27 on Data Protection (RGPD) and the organic law 3 /2018, of December 5, protection of personal data and guarantee of digital rights. The database is kept by the principal investigator and the research team in an Excel format, protected by password access. An anonymized database was used for the analysis. Before carrying out the survey, online informed consent had to be completed, accepted, and signed.

### Variables

The main variables were the following: sociodemographic factors (gender, age, having children under 15 years of age, level of education); sociocultural factors (beliefs, culture, family); vaccine refusal (if any vaccine had been previously refused, opinion about vaccines); network search (last year of search, if it was about COVID-19); and the effect of the COVID-19 pandemic on the intention to get vaccinated against COVID-19 and on the doubts and decision about routinely recommended vaccines.

### Statistical Analysis

All the variables are described using absolute and relative frequencies (numbers and percentages of positive and negative responses). The main variables were related to the effect of the COVID-19 pandemic on the following aspects: intention to get vaccinated against COVID-19 as well as doubts and decision about other routinely recommended vaccines. To determine if there are sociocultural differences related to these main variables, the different groups were compared using chi-square tests. The effects of all the variables (dependent and independent) were evaluated on each dependent variable. Statistical significance was established at *P*<.05. Statistical analyses were performed with R software version 4.1.2.

## Results

### Sample Overview

A total of 1188 questionnaires were collected and included in the study. In the sample, 73.8% (870/1179) of the respondents were women, 47.0% (558/1187) had sons or daughters under the age of 15 years, and 71.7% (852/1188) had studied at university. Regarding vaccination, 16.3% (193/1187) of the participants stated that they had refused a vaccine on some occasion, 76.3% (907/1188) totally agreed with vaccines, 1.9% (23/1188) were indifferent, and 3.5% (41/1188) and 1.2% (14/1188) slightly or totally disagreed with vaccination, respectively. The median time since the last internet search on vaccination was 1 year. Moreover 25.3% (301/1188) of all respondents claimed to have consulted on social networks about COVID-19 vaccines, representing 84% (301/360) of those who answered the question Table 1 contains the description of the sample.

**Table 1:** Sample overview (n=1188).

Variables			Results, n (%)
**Sociodemographic variables**			
	**Sex^a^**		
		Men	309 (26.2)
		Women	870 (73.8)
	**Age (years)^b^**		
		<25	93 (7.8)
		26-30	64 (5.4)
		31-39	232 (19.6)
		40-49	347 (29.2)
		50-59	307 (25.9)
		≥60	144 (12.1)
	**Do you have daughters or sons aged 14 years or under?^b^**		
		Yes	558 (47.0)
		No	629 (53.0)
	**What is your education level?**		
		Without or with incomplete primary education	4 (0.3)
		Primary	25 (2.1)
		Secondary	48 (4.1)
		Bachelor’s degree	77 (6.5)
		Vocational training	182 (15.3)
		University	852 (71.7)
**Sociocultural environment**			
	**Your religious beliefs are:^c^**		
		In favor of vaccination	439 (37.1)
		Against vaccination	6 (0.5)
		Neutral	192 (16.2)
		I am not a believer	447 (37.8)
		I do not know	100 (8.4)
	**Your culture is:^d^**		
		In favor of vaccination	1040 (87.8)
		Against vaccination	10 (0.8)
		Neutral	89 (7.5)
		I do not know	46 (3.9)
	**Your family is:^d^**		
		In favor of vaccination	1089 (91.9)
		Against vaccination	25 (2.1)
		Neutral	51 (4.3)
		I do not know	20 (1.7)
**Vaccine refusal**			
	**Have you ever refused any vaccine?^b^**		
		Yes	193 (16.3)
		No	994 (83.7)
	**Regarding vaccines**		
		Totally in agreement	907 (76.3)
		Slightly in agreement	203 (17.1)
		Indifferent	23 (1.9)
		Slightly in disagreement	41 (3.5)
		Totally in disagreement	14 (1.2)
**Consultation on social networks**			
	Years from the last search you did on social networks^e,f^		1.00 (0.00-1.00)
	**Was the search related to COVID-19?**		
		Yes	301 (25.3)
		No	59 (4.9)
		Not applicable	828 (69.7)
**Questions about COVID-19**			
	**As a result of the COVID-19 pandemic, will you get vaccinated against COVID-19 when you are asked?^g^**		
		Yes	1069 (90.8)
		No	108 (9.2)
	**As a result of the COVID-19 pandemic, have your doubts about vaccination increased?^h^**		
		Yes	359 (30.3)
		No	824 (69.7)
	**As a result of the COVID-19 pandemic, have you changed your decision about other vaccines?^i^**		
		Yes	154 (13.0)
		No	1028 (87.0)

^a^n=1179.

^b^n=1187.

^c^n=1184.

^d^n=1185.

^e^median (IQR).

^f^n=330.

^g^n= 1177.

^h^n=1183.

^i^n=1182.

### Intention of Getting Vaccinated Against COVID-19

Of the people surveyed, 90.8% (1068/1177) stated that, as a result of the COVID-19 pandemic, they would get vaccinated against COVID-19 when asked, while 9.2% (108/1188) stated the opposite.

There was a significantly greater intention to get vaccinated among women, people older than 50 years of age, and people without children 15 years of age or younger. Moreover, religious beliefs, culture, or family environment favorable to vaccination had a significant positive association with getting vaccinated. Participants who had not previously rejected other vaccines and who were totally in favor of vaccines were significantly more likely to get vaccinated. Also, a significantly greater intention to get vaccinated against COVID-19 was found among people whose doubts about vaccination did not increase and who did not change their decision about the other vaccines as a result of the pandemic (Table 2).

In particular, women with children younger than 15 years of age were less ready to get vaccinated against COVID-19, and their doubts about vaccination significantly increased as a result of the COVID-19 pandemic (Table 3).

**Table 2:** Intention of getting vaccinated against COVID-19 (n=1177).

Characteristics			As a result of the COVID-19 pandemic, would you get vaccinated against COVID-19 when you are asked?		Overall *P* value
			Yes (n=1068), n (%)	No (n=108), n (%)	
**Sociodemographic variables**					
	**Sex**				.02
		Men	269 (25.4)	39 (36.1)	
		Women	790 (74.6)	69 (63.9)	
	**Age (years)**				.02
		<25	80 (7.5)	13 (12.1)	
		26-30	53 (5.0)	11 (10.3)	
		31-39	204 (19.1)	24 (22.4)	
		40-49	310 (29.0)	32 (29.9)	
		50-59	286 (26.8)	19 (17.8)	
		≥60	135 (12.6)	8 (7.5)	
	**Do you have daughters or sons aged 14 years or under?**				.009
		Yes	487 (45.6)	64 (59.3)	
		No	580 (54.4)	44 (40.7)	
	**What is your education level?**				.25
		Without or with incomplete primary education	4 (0.4)	0 (0)	
		Primary	23 (2.1)	2 (1.9)	
		Secondary	39 (3.6)	9 (8.3)	
		Bachelor’s degree	70 (6.6)	6 (5.6)	
		Vocational training	161 (15.1)	20 (18.5)	
		University	771 (72.2)	71 (65.7)	
	**University studies**				.19
		No	297 (27.8)	37 (34.3)	
		Yes	771 (72.2)	71 (65.7)	
**Sociocultural environment**					
	**Your religious beliefs are:**				<.001
		In favor of vaccination	420 (39.5)	14 (13.0)	
		Against vaccination	2 (0.2)	4 (3.7)	
		Neutral	158 (14.8)	33 (30.5)	
		I am not a believer	389 (36.6)	54 (50.0)	
		I do not know	95 (8.9)	3 (2.8)	
	**Your culture is:**				<.001
		In favor of vaccination	971 (91.1)	59 (55.1)	
		Against vaccination	3 (0.3)	7 (6.6)	
		Neutral	57 (5.3)	32 (29.9)	
		I do not know	35 (3.3)	9 (8.4)	
	**Your family is:**				<.001
		In favor of vaccination	1015 (95.1)	64 (60.4)	
		Against vaccination	11 (1.0)	13 (12.2)	
		Neutral	31 (2.9)	20 (18.9)	
		I do not know	10 (1.0)	9 (8.5)	
**Vaccine refusal**					
	**Have you ever refused any vaccine?**				<.001
		Yes	142 (13.3)	49 (45.8)	
		No	926 (86.7)	58 (54.2)	
	**Regarding vaccines:**				<.001
		Totally in agreement	881 (82.5)	21 (19.5)	
		Slightly in agreement	166 (15.6)	32 (29.6)	
		Indifferent	10 (0.9)	12 (11.1)	
		Slightly in disagreement	10 (0.9)	30 (27.8)	
		Totally in disagreement	1 (0.1)	13 (12.0)	
**Consultation on social networks**					
	Years from the last search you did on social networks^a^		0.8 (1.6)	0.7 (0.5)	.33
	**Was the search related to COVID-19?**				.14
		Yes	266 (84.7)	32 (74.4)	
		No	48 (15.3)	11 (25.6)	
**Questions about COVID-19**					
	**As a result of the COVID-19 pandemic, have your doubts about vaccination increased?**				<.001
		Yes	274 (25.7)	77 (71.3)	
		No	792 (74.3)	31 (28.7)	
	**As a result of the COVID-19 pandemic, have you changed your decision about vaccines?**				<.001
		Yes	104 (9.8)	45 (41.7)	
		No	960 (90.2)	63 (58.3)	

^a^median (IQR).

**Table 3:** Comparison of results obtained to the questions according to gender.

Characteristics		Has sons or daughters ≤14 years old, n (%)		Does not have sons nor daughters ≤14 years old		*P* value
		Men	Women	Men	Women	
**As a result of the COVID-19 pandemic, will you get vaccinated against the COVID-19 vaccine when proposed?**						
	Yes	104 (38.7)	382 (48.4)	165 (61.3)	407 (51.6)	.18
	No	20 (51.3)	44 (63.8)	19 (48.7)	25 (36.2)	.02
**As a result of the COVID-19 pandemic, have your doubts about vaccination increased?**						
	Yes	42 (52.5)	155 (56.0)	38 (47.5)	122 (44.0)	.02
	No	82 (36.1)	275 (46.8)	145 (63.9)	313 (53.2)	.01
**As a result of the COVID-19 pandemic, have you changed your decision about vaccines?**						
	Yes	155 (80.3)	155 (56.0)	38 (19.7)	122 (44.0)	.08
	No	82 (36.1)	275 (46.8)	145 (63.9)	313 (53.2)	>.99

### Increasing Doubts on Routinely Recommended Vaccination

Of the respondents, 30.3% (359/1183) stated that their doubts regarding vaccination in general had increased as a result of the COVID-19 pandemic. There were no statistical differences in such a response with respect to gender, university studies, or searching on social networks. A significantly higher percentage of participants with increased doubts were younger than 50 years of age or had children younger than 15 years of age, and their beliefs, culture, or family were against vaccination. Moreover, participants with increased doubts had sometimes rejected vaccines; they were not totally in favor of vaccines and reported that the pandemic had changed their decision about vaccines (Table 4).

**Table 4:** Doubts on routinely recommended vaccination (n=1183).

Characteristics			As a result of the COVID-19 pandemic, have your doubts about vaccination increased?		Overall *P* value
			Yes (n=359), n (%)	No (n=824), n (%)	
**Sociodemographic variables**					
	**Sex**				.057
		Men	80 (22.3)	227 (27.9)	
		Women	278 (77.7)	588 (72.1)	
	**Age (years)**				<.001
		<25	27 (7.5)	66 (8.0)	
		26-30	27 (7.5)	37 (4.5)	
		31-39	85 (23.8)	146 (17.7)	
		40-49	116 (32.4)	230 (28.0)	
		50-59	74 (20.7)	231 (28.1)	
		≥60	29 (8.1)	113 (13.7)	
	**Do you have daughters or sons aged 14 years or under?**				<.001
		Yes	197 (55.0)	358 (43.5)	
		No	161 (45.0)	465 (56.5)	
	**What is your education level?**				.004
		Without or with incomplete primary education	0 (0.0)	4 (0.5)	
		Primary	6 (1.7)	18 (2.2)	
		Secondary	22 (6.1)	25 (3.0)	
		Bachelor’s degree	16 (4.5)	60 (7.3)	
		Vocational training	69 (19.2)	113 (13.7)	
		University	246 (68.5)	603 (73.3)	
	**University studies**				.11
		No	113 (31.5)	220 (26.7)	
		Yes	246 (68.5)	603 (73.3)	
**Sociocultural environment**					
	**Your religious beliefs are:**				.001
		In favor of vaccination	111 (30.9)	324 (39.6)	
		Against vaccination	5 (1.4)	1 (0.1)	
		Neutral	74 (20.6)	118 (14.4)	
		I am not a believer	134 (37.3)	312 (38.1)	
		I do not know	35 (9.8)	64 (7.8)	
	**Your culture is:**				<.001
		In favor of vaccination	277 (77.4)	757 (92.2)	
		Against vaccination	7 (1.9)	3 (0.3)	
		Neutral	49 (13.7)	40 (4.9)	
		I do not know	25 (7.0)	21 (2.6)	
	**Your family is:**				<.001
		In favor of vaccination	296 (82.9)	787 (95.8)	
		Against vaccination	19 (5.3)	6 (0.7)	
		Neutral	31 (8.7)	20 (2.4)	
		I do not know	11 (3.1)	9 (1.1)	
**Vaccine refusal**					
	**Have you ever refused any vaccine?**				<.001
		Yes	85 (23.8)	108 (13.1)	
		No	272 (76.2)	716 (86.9)	
	**Regarding vaccines:**				<.001
		Totally in agreement	176 (49.0)	727 (88.3)	
		Slightly in agreement	126 (35.1)	75 (9.1)	
		Indifferent	16 (4.5)	7 (0.9)	
		Slightly in disagreement	32 (8.9)	9 (1.1)	
		Totally in disagreement	9 (2.5)	5 (0.6)	
**Consultation on social networks**					
	Years from the last search you did on social networks^a^		0.7 (1.0)	0.8 (1.7)	.41
	**Was the search related to COVID-19?**				.55
		Yes	104 (81.9)	197 (84.9)	
		No	23 (18.1)	35 (15.1)	
**Questions about COVID-19**					
	**As a result of the COVID-19 pandemic, will you get vaccinated against COVID-19 when you are asked?**				<.001
		Yes	274 (78.1)	792 (96.2)	
		No	77 (21.9)	31 (3.8)	
	**As a result of the COVID-19 pandemic, have you changed your decision about other vaccines?**				<.001
		Yes	122 (34.0)	32 (3.9)	
		No	237 (66.0)	788 (96.1)	

^a^median (IQR).

### Variation of the Intention to Get Vaccinated With Routinely Recommended Vaccines

Of the respondents to the survey, 13.0% (154/1182) stated that, as a result of the COVID-19 pandemic, their decision about recommended vaccines had changed. In this case, there were no statistically significant differences regarding gender, age, having children younger than 15 years of age, educational level, religious beliefs, or searching on networks. Most of the people who did not change their opinion about vaccines were totally in favor of vaccines or their culture or family was (Table 5).

**Table 5:** Variation of the intention to get vaccinated with routinely recommended vaccines (n=1182).

Characteristics			As a result of the COVID-19 pandemic, have you changed your decision about vaccines?		Overall *P* value
			Yes (n=154), n (%)	No (n=1028), n (%)	
**Sociodemographic variables**					
	**Sex**				.053
		Men	30 (19.5)	277 (27.2)	
		Women	124 (80.5)	741 (72.8)	
	**Age (years)**				.09
		<25	15 (9.8)	78 (7.6)	
		26-30	14 (9.1)	50 (4.9)	
		31-39	36 (23.6)	194 (18.9)	
		40-49	41 (26.8)	304 (29.6)	
		50-59	33 (21.6)	272 (26.5)	
		≥60	14 (9.1)	129 (12.5)	
	**Do you have daughters or sons aged 14 years or under?**				.33
		Yes	78 (51.0)	476 (46.3)	
		No	75 (49.0)	551 (53.7)	
	**What is your education level?**				.052
		Without or with incomplete primary education	0 (0)	4 (0.4)	
		Primary	5 (3.2)	20 (2.0)	
		Secondary	11 (7.2)	36 (3.5)	
		Bachelor’s degree	11 (7.2)	66 (6.4)	
		Vocational training	31 (20.1)	151 (14.7)	
		University	96 (62.3)	750 (73.0)	
**Sociocultural environment**					
	**Your religious beliefs are:**				.12
		In favor of vaccination	46 (29.9)	388 (37.9)	
		Against vaccination	2 (1.3)	4 (0.4)	
		Neutral	32 (20.8)	160 (15.7)	
		I am not a believer	61 (39.6)	385 (37.6)	
		I do not know	13 (8.4)	86 (8.4)	
	**Your culture is:**				<.001
		In favor of vaccination	117 (76.0)	916 (89.5)	
		Against vaccination	2 (1.3)	8 (0.8)	
		Neutral	22 (14.3)	67 (6.5)	
		I do not know	13 (8.4)	33 (3.2)	
	**Your family is:**				<.001
		In favor of vaccination	122 (80.3)	961 (93.6)	
		Against vaccination	10 (6.6)	15 (1.5)	
		Neutral	11 (7.2)	40 (3.9)	
		I do not know	9 (5.9)	11 (1.0)	
**Vaccine refusal**					
	**Have you ever refused any vaccine?**				<.001
		Yes	43 (27.9)	149 (14.5)	
		No	111 (72.1)	877 (85.5)	
	**Regarding vaccines:**				<.001
		Totally in agreement	60 (39.0)	842 (81.9)	
		Slightly in agreement	64 (41.6)	138 (13.4)	
		Indifferent	7 (4.5)	16 (1.6)	
		Slightly in disagreement	20 (13.0)	21 (2.0)	
		Totally in disagreement	3 (1.9)	11 (1.1)	
**Consultation on social networks**					
	Years from the last search you did in social networks^a^		0.6 (0.5)	0.8 (1.6)	.10
	**Was the search related to COVID-19?**				.20
		Yes	43 (76.8)	257 (84.8)	
		No	13 (23.2)	46 (15.2)	
**Questions about COVID-19**					
	**As a result of the COVID-19 pandemic, will you get vaccinated against COVID-19 when you are asked?**				<.001
		Yes	104 (69.8)	960 (93.8)	
		No	45 (30.2)	63 (6.2)	
	**As a result of the COVID-19 pandemic, have your doubts about vaccination increased?**				<.001
		Yes	122 (79.2)	237 (23.1)	
		No	32 (20.8)	788 (76.9)	

^a^median (IQR).

## Discussion

The objective of our study was to evaluate the impact of the pandemic on public opinion about the decision to get vaccinated against COVID-19, doubts about routinely recommended vaccinations, and the final decision about vaccination. Also, we wanted to determine if sociodemographic and socioeconomic factors influenced decision-making about vaccination.

Of the surveyed population of residents of Catalonia aged at least 18 years in 2021, 90.8% showed a predisposition to get vaccinated against COVID-19, a lower percentage than that published in a serial survey conducted during the COSMO-Spain study promoted by the WHO (round December 9, 2021). According to the latter, 96% of the surveyed population stated they had received at least one dose of COVID-19 vaccine [23]. Similarly, in December 2021, the barometer of the Spanish Centre for Sociological Research showed that 96.5% of people surveyed had already been vaccinated against COVID-19, 3.2% had not, and 0.3% did not respond. Of the latter, 59.5% were not willing to be vaccinated, 4% had doubts, 3.1% would only do so under certain conditions, 21.7% would be vaccinated, and the rest did not respond [24]. Data from 2020 published by the Ministry of Health showed a primary vaccination coverage of 97% and a coverage for the 2 doses of the measles, mumps, and rubella (MMR) vaccine of 94.2% [25]. Finally, recent data showed coverage of the vaccine against COVID-19 of 90.4% and 85.4% of the population over 12 years of age in Spain and Catalonia, respectively [19,20]. This represented good vaccination coverage in Spain for both routinely recommended vaccines and the vaccine against COVID and exceeded the Spanish coverage for the 2020-2021 flu campaign, which was 67.7% for people older than 65 years of age and 72.0% for people older than 75 years of age [25].

In our study, 30.3% of people surveyed stated that, as a result of the COVID-19 pandemic, their doubts regarding recommended vaccinations had increased. Such doubts could imply a decrease in vaccination rates in general, such as the primary vaccination coverage, which is currently 97%. This effect could be mitigated by the evolution of the predisposition to vaccination during the pandemic. Indeed, the willingness of the Spanish population to get vaccinated against COVID-19 has been evolving with the progress of the vaccination campaign, as shown by the serial surveys carried out by the COSMO-Spain study. In this study, in the surveys prior to the start of the vaccination campaign, the percentage of people willing to get vaccinated against COVID-19 was 39% in November 2020. In the December 2021 survey, 96% of those surveyed reported having received some dose of the vaccine, and only 2% said they did not want to be vaccinated [23].

A study conducted in the United States during the pandemic also found that parental doubts about vaccinating children and the perception of risk about vaccines had increased. However, these doubts did not translate into less intention to administer routine vaccines to children [26]. This observation coincides with our results showing an increase in doubts about vaccination but an unchanged decision about vaccines by 87.0% of this population. However, 13.0% of the participants in the survey stated that, as a result of the COVID-19 pandemic, their decision on recommended vaccines had changed. This last group is related to antivaccination profiles, which could make it difficult for health professionals to maintain high vaccination coverage.

As for the sociodemographic variables, in contrast to other articles, we detected a greater predisposition to get vaccinated against COVID-19 or other vaccine-preventable diseases in women than in men [27,28]. Interestingly, as a result of the pandemic, women with children younger than 15 years of age were less likely to get vaccinated against COVID-19. Similarly, a review by Joshi et al [6] in 2021 showed that women and parents manifest less acceptance of the COVID-19 vaccine, which is why improving confidence in vaccines is recommended, especially in mothers. Other studies conducted with the UK and US populations indicated that women with young children are more concerned about vaccinating their children [28,29]. This could explain the high participation of women in our study, and we could deduce that, at certain socioeconomic levels, decision-making about the vaccination of children could be predominantly made by mothers. This observation is in agreement with data published by the Spanish Ministry of Health and Social Policy in 2009, showing greater maternal involvement in the care of other people in the family; moreover, in 2020, the European Commission ratified this situation [30,31]. In the same line, the dissemination through mommy blogs of the ideology of intensive motherhood, which is a cultural model of appropriate childrearing according to which mothers should unselfishly make a tremendous investment in their child, maintains the persistence of gender inequality and allows the dissemination of erroneous information about vaccines [32,33].

In agreement with other studies, we found a greater predisposition to get vaccinated and fewer doubts about vaccination as a result of the COVID-19 pandemic among older adults. Indeed, it is argued that older people are more likely to get vaccinated than younger people, since they are more concerned about their health and are more susceptible to getting sick [3,27,34,35].

Despite the high participation of people who had completed university studies, no relationship was found with attitudes for or against vaccination. Some studies have shown that the higher the level of education, the greater the acceptance of vaccines and the predisposition to prioritize vaccination [36-40]. On the contrary, there are also studies that found that sociodemographic levels do not influence the decision to vaccinate [41] or that a higher level of education coincides with greater doubts about vaccination [42].

Our study shows that cultural, religious, and family beliefs are associated with doubts and decision about vaccination, with a greater predisposition to get vaccinated against COVID-19 if the sociocultural environment is provaccine. These results support data published in previous studies [43,44].

We found that people who were not predisposed to get vaccinated against COVID-19 also stated that their doubts about vaccination increased as a result of the pandemic or reported having refused vaccines sometimes. This could indicate that there may be other factors, apart from sociodemographic and sociocultural factors, that can influence vaccine hesitancy: accessibility and cost, personal responsibility and risk perception, precautionary measures taken to vaccinate people, trust in health authorities and in vaccines, safety and efficacy of a new vaccine, and lack of information or misinformation about vaccines [3,6,44,45]. These factors cannot be interpreted either causally or independently of each other. It is much more plausible that several of the factors interrelate in a complex and dynamic way to influence individuals, confirming the WHO 3C Model (confidence, complacency, and convenience) of the factors influencing the decision to get vaccinated [3,46].

There is extensive literature showing that information from social networks can be a source of vaccine hesitancy; however, in this study, it was not possible to establish this association since only one-quarter of the respondents consulted social networks about COVID-19 vaccines [2,4,11-13]. This low level of searching on social networks could be explained by the results of other studies, such as the study by Hunt [47], which showed that people passively receive information from networks and are not aware of it [48], such as being a follower of mommy blogs and passively receiving erroneous information about vaccines [32]. However, we think that social networks provide an opportunity to directly communicate medical information to the public and that health systems should work on building disease detection and surveillance systems through the monitoring of social networks [49].

One of the limitations of this study is recruitment bias since people without access to the online survey could not participate. However, 97% of Catalan households have internet access, and 70.4% of Catalan internet were users of social networks in 2020 [50,51]. Another limitation is the impossibility of detecting if anyone repeated the survey; however, it was expected that few respondents would repeat it and therefore it would not influence the final result of the total sample. In the population surveyed, there was a predominance of women and people with a university education. This fact could result in not being fully representative of the Catalan population and could limit the interpretation and generalization of our study. The difficulty of reaching the antivaccine population using this survey was also known due to the difficulties in interacting with these groups. Therefore, to limit eventual bias deriving from this, in the sample size calculation, we considered that the proportion of antivaccine responses would be much lower than the provaccine responses.

### Conclusions

The population studied was predominantly in favor of vaccination, although there were high percentages of rejection specifically of vaccination against COVID-19. As a result of the pandemic, we detected an increase in doubts about vaccines. Although the final decision about vaccination did not primarily change, some of the respondents did change their opinion about routine vaccinations. The seed of doubt about vaccines and the changes in opinion generated as a result of the pandemic can be worrying for maintaining high vaccination coverage and could make the work of health professionals difficult.

This study has improved the knowledge on opinions about vaccines, and this will allow optimizing of the approach to vaccination in certain population groups with greater hesitation.

## Appendix

Multimedia Appendix 1 MC-MUVA work group.

## Abbreviations

MMR: measles, mumps, and rubella
REDCap: Research Electronic Data Capture
WHO: World Health Organization

## Data Availability Statement

The data are hosted on the research team’s internal servers and will be provided under reasonable request.
