# Supplementary material for: Effects of the COVID-19 Pandemic on the Decision and Doubts About Vaccination in Catalonia: Online Cross-sectional Questionnaire
Source: JMIR Form Res. 2023 Mar 6;7:e41799. doi: 10.2196/41799 (PMC9994466; doi:10.2196/41799)
Supplement: Multimedia Appendix 1 [file formative_v7i1e41799_app1.docx]

**MC-MUVA work group**

Rebecca Oglesby, Merce Font Arbó, Lidia Sanz Borrell, Mireia Biosca Pàmies, Marta Pifarre Ortiz, Sara Martí Martí, Daniel Gros Esteban, Laura Guix Cliville, Noelia Diaz Charles, Silvia Prado Muñoz, Susana Perez Osuna, Mercè Giribet Folch, Magda Riera Veciana, Carles Gatius Tonda, Laura Seoane Barbosa, Pilar Serra Solans, Míriam Poblet, M. José Castañ Castillo, Elena Alcover Bloch, Anna Castan, Magda Barberà Farré, Sara Serra Font, Mònica Martinez, Imma Caubet Busquet, Maria Planella Cornudella, Gema Terrer Manrique, Raul Morales, Anna Gatell Carbo, Carme Farran Balcells,Carmen Gómez Seara, Viktoriya Atroshchenko Shushko, Margaret Creus Verni, Veronida Mª Karcz, Judith Palacin Aguilà, Joana Garcia Hinojosa, Mihaela Cozar, Rosa Aran Padullés, Ramón Capdevila Bert,Montse Crespo Pons, Maria Jose Lara Tostado, Isabel Segarra Solanes, Enmanuel Aneudi Estevez Genao, Raquel Plasencia Atienza, Maria Chiné Segura, Meritxell Pelegrí Romeo, Montse Farran Jové, Carme Trilla Felis, Rubén Lopez Ruiz, Iratxe Olabegoya Estrela,Roser Racero Maire, Sonia Asensio, Merce Lozano Vergara, Mercè Cortés Lladó, Àngels Font Anglada, Sara Borrat Padrosa, Maria Payan Sala,Bárbara Montilla Cabrera, Samanta Barisonzi Zambrano, Almudena Sanchez, Cristina Casademont, Pyrene Martinez, Anna M. Ristol Perxés, Irene Gómez i Pérez, Jaume Miquel Salsas
